# LncRNA-mRNA modules involved in goat rumen development: Insights from genome-wide transcriptome profiling

**DOI:** 10.3389/fphys.2022.979121

**Published:** 2022-08-24

**Authors:** Tao Zhong, Juan Zhao, Siyuan Zhan, Linjie Wang, Jiaxue Cao, Dinghui Dai, Jiazhong Guo, Li Li, Hongping Zhang, Lili Niu

**Affiliations:** Farm Animal Genetic Resources Exploration and Innovation Key Laboratory of Sichuan Province, College of Animal Science and Technology, Sichuan Agricultural University, Chengdu, China

**Keywords:** Goat, rumen, transcriptome, lncRNA, mRNA, different expression, pathway

## Abstract

The rumen is an essential digestive and absorption organ of ruminants. During fetal life, lactation, and post-weaning period, goat rumen undergoes drastic morphological and metabolic-functional changes triggered by potential regulated genes and non-coding RNA molecules. As the essential regulatory factors, long non-coding RNAs (lncRNAs) have vital functions in various biological activities. However, their roles during rumen development are still poorly explored in ruminants. To explore the genome-wide expression profiles of lncRNAs and mRNAs in the goat rumens, we generated 5,007 lncRNAs and 19,738 mRNAs identified during the fetal and prepubertal stages by the high-throughput RNA sequencing. Notably, 365 lncRNAs and 2,877 mRNAs were considered to be differentially expressed. The weighted gene co-expression network analysis and functional analysis were performed to explore the regulatory roles of those differentially expressed molecules. The *cis-*and *trans*-target genes of differently expressed lncRNAs were enriched for pathways related to focal adhesion, cGMP-PKG signaling pathway, alpha-linolenic acid metabolism, arachidonic acid metabolism, and fat digestion and absorption. Gene Ontology and the Kyoto Encyclopedia of Genes and Genomes analyses showed that the differently expressed genes mainly participated in mitotic cytokinesis, desmosome, fatty acid degradation, cell adhesion molecules, and fatty acid metabolism. The prediction of lncRNA-mRNA interaction networks further revealed transcripts potentially involved in rumen development. The present study profiles a global overview of lncRNAs and mRNAs during rumen development. Our findings provide valuable resources for genetic regulation and molecular mechanisms of rumen development in ruminants.

## Introduction

The rumen is the vital digestion and absorption organ, and its development is closely related to weaning and young ruminant performance. As early as 35 days of prenatal life, goat rumen was generated from the primitive gastric tube (García et al., 2012). Subsequently, ruminal papillae were observed at 46 days, protruded from the internal epithelial layer at 50 days of prenatal life, and were leaf-shaped with surface keratinization before birth (García et al., 2012). Nevertheless, the rumen epithelium is physiologically and functionally incomplete in neonatal ruminants (Diao et al., 2019). During the transition from non-rumination (before weaning) to the rumination stage (after weaning), the morphology and structure of the rumen epithelium have significant changes (Jiao et al., 2016; Khan et al., 2016). The nutrient supply of ruminants also alters from the high-fat milk diets in the non-ruminant period to forage-based diets during the rumination stage. Since voluntary forage intake, roughage stimulates the rapid development of the young rumens and microbial colonization therein (Lane et al., 2000). Short-chain fatty acids, especially butyric acid generated by feed fermentation, could further stimulate the growth of rumen papillae (Carballo et al., 2019). Many factors have been verified to affect the ontogenesis of the rumen, such as genetics (Li F et al., 2019), neuroendocrine (Franco et al., 2014), the age of weaning (Rybak-Wolf et al., 2015; Carballo et al., 2019), composition and characteristics of solid feed (Huang et al., 2010; Berends et al., 2012), dietary nutrition and neutral detergent fiber levels (Diao et al., 2019; Xie et al., 2020). To date, the dynamic changes of the potential genes related to rumen development are poorly understood.

Previous studies have revealed that most economic traits are regulated by candidate mRNAs and complex biological networks of non-coding RNAs in livestock (Zhang et al., 2018; Muret et al., 2019; Zhao et al., 2021). In 2020, Li et al. (2020) found that most (77.65%) of the rumen core genes were enriched with ruminant-specific non-coding conservative sequences. Among them, long non-coding RNAs (lncRNAs) specifically participate in many critical regulatory processes at the epigenetic, transcription, post-transcriptional, translation, and post-translational levels and also play vital roles in various cellular processes (Wei et al., 2017; Wang et al., 2018; Vafadar et al., 2019; Zhang et al., 2019). Recent studies have confirmed that lncRNAs are associated with brown fat formation (Alvarez-Dominguez et al., 2015), skeletal muscle development (Sun et al., 2016), and cashmere growth (Wang et al., 2017). In 2020, He et al. (2020) proposed that LncWNT3-IT can promote the proliferation of goat Sertoli cells by positively regulating the expression of the *WNT3* gene. In addition, lncRNAs are also involved in the regulation of diseases. For instance, lncRNA-MEG3 positively regulated the expression of the *TLR4* gene through miR-210, participating in the inflammatory response and apoptosis in porcine alveolar macrophages infected with Haemophilus parasuis (Yin et al., 2021). In the mice model of cardiac hypertrophy, the lncRNA-XIST was verified to be a necessary regulator of the formation of cardiac hypertrophy *via* the miR-101/TLR2 axis in both *in vivo* and *in vitro* assays (Xiao et al., 2019). Thus, we proposed that lncRNA plays an essential role in the rumen development of goats.

As previously explored, *IGF-1* can up-regulate the expression of cyclin D1 through the Ras/Raf/MEK/ERK signaling pathway and regulate the proliferation of rumen epithelial cells (Lu et al., 2013). The reduced expression of *IGFBP* may promote the proliferation of rumen epithelial cells by promoting *IGF-1* (Nishihara et al., 2020). Several genes have been revealed to be associated with the growth of ruminal papillae, such as sodium butyrate infusion regulates monocarboxylate transporter isoform 1 (*MCT1*), 3-hydroxy-3-methylglutaryl-CoA synthase isoform 2 (*HMGCS2*), 3-hydroxy-3-methylglutaryl-CoA lyase (*HMGCL*) and sodium/proton exchanger isoform 2 (*NHE2*) (Sun et al., 2018; Liu et al., 2019). miR-122 was differentially expressed in grass-fed and grain-fed Angus cattle rumen tissue and may affect rumen function by targeting OCLN and RBM47 genes related to gastrointestinal function. miR-655 was detected only in the grain-fed group, and its target was significantly enriched in insulin and TGF-β signaling pathways, which may synergistically regulate rumen function (Li Y et al., 2019). It has been reported that members of the *bta*-miR-143, miR-29b, miR-145, miR-493, miR-26a, and miR-199 families may be critical regulators of rumen development of calves (Do et al., 2019). In our previous studies, some candidate miRNAs and circRNAs had been identified to be related to rumen development (Zhong et al., 2017; Zhong et al., 2020; Zhong et al., 2022). miR-148a-3p continued to be highly expressed in fetuses and targets *QKI*, suggesting that miR-148a-3p was involved in the development of early rumen epithelial cells through targeting *QKI* (Zhong et al., 2020). However, the biological functions of lncRNAs and mRNAs during rumen development have not been systematically explored in goats. In the present study, we captured the whole genome-wide expression profiling of lncRNAs and mRNAs involved in goat rumens during ontogenesis. The functional analyses were performed to reveal the regulatory modules of lncRNA-mRNA molecules at the fetal stage (F60 and F135), before weaning (BW30), and after weaning (AW150) stages, respectively.

## Materials and methods

### Tissues collection, RNA preparation, and sequencing

The Jianzhou big-ear goats were used in this study, characterized by fast growth, crude feed tolerance, slaughter rate, and adaptation to subtropical climate. The twelve rumens were separated from the fetuses at 60 and 135 days of gestation (F60 and F135 groups, *n* = 3), the 30-day-old goat kids (before weaning, BW30, *n* = 3), and the 150-day-old prepubertal goats (after weaning, AW150, *n* = 3), respectively. Small pieces of tissues were collected from the dorsal and ventral sac of the rumen. The detailed information about animal feeding and management and the sampling procedure was mentioned in our previous study (Zhong et al., 2022). Furthermore, the reticulum, omasum, and abomasum were also collected to compare the differences in the ontogenesis of the stomach. RNA was isolated by the TRIzol reagent (Invitrogen, CA, United States) and used to construct the high strand-specificity libraries. Subsequently, the twelve libraries were sequenced using the Illumina Hiseq 4,000 platform (Illumina, San Diego, CA, United States).

### Slice processing and morphometric analysis

A small piece of each tissue was dissected and fixed in 4% paraformaldehyde (Beyotime Biotech Inc., Shanghai, China). After that, the tissues were routinely dehydrated in increased grades of alcohol (75% alcohol for 4 h, 85% alcohol for 2 h, 95% alcohol for 1 h, 100% alcohol for 0.5 h), and immersed in xylene, then embedded in paraffin. The specimen was transversely cut by a Leica RM2016 Rotary Slicer (Leica Microsystems, Wetzlar, Germany) and stained with Haematoxylin and Eosin. The slices were viewed by an Olympus BX-53 fluorescence microscope with a DP-80 digital camera (Olympus Corporation, Tokyo, Japan). Fifty measured values, obtained by a cellSens Standard 1.16 imaging system (Olympus), were used to estimate the height of epithelium, lamina propria, mucous membrane, and tunica muscularis for each specimen.

### Quality control and lncRNAs identification

The raw data (Fastq format) were processed using in-house Perl scripts to remove the adapter sequences, reads with over 10% N sequences, and low-quality reads, in which the number of bases with a quality value Q ≤ 10 was > 50%. Reads were mapped to the reference genome ARS1 using HISAT2 (version 2.0.4) (Kim et al., 2005) and assembled using StringTie (version 1.3.1) (Pertea et al., 2015). After mapping with the ARS1 goat reference genome, a strict filtering pipeline was used to identify the potential lncRNA transcripts following the four steps: 1) transcripts annotated as “u,” “i,” and “x,” representing long intergenic lncRNAs, intronic lncRNAs, and antisense lncRNAs, were retained, 2) transcripts with single exon and length less 200 bp were eliminated, 3) transcripts with the fragments per kilobase of transcript per millions mapped reads (FPKM) greater than 0.1 in any of the four groups were retained, 4) only the transcripts without coding potential, as predicted by the coding-non-coding index (CNCI <0, v2) (Sun et al., 2013), coding potential calculator (CPC <0, v1.3) (Kong et al., 2007), protein folding domain database (Pfam v1.3, E-value ≤10^−5^) (El-Gebali et al., 2019), and coding potential assessing tool (CPAT <0.364) (Wang et al., 2013), were denoted as lncRNA candidates.

### Expression, PCA, and weighted gene co-expression network analysis analyses

The expression levels of lncRNAs and mRNAs were normalized by Cuffdiff v2.1.1. The differently expressed lncRNAs (DELs) and differently expressed genes (DEGs) were identified using the DE Seq2 package with the threshold (false discovery rate (FDR) < 0.05 and |log2 (Fold Change)| > 1) (Love et al., 2014). The PCA plots and weighted gene co-expression network analysis were performed using the ggplot2 and weighted gene co-expression network analysis (WGCNA) packages in R v4.2.0 (Langfelder and Horvath, 2008). First, the Hclust function was used for hierarchical clustering to exclude abnormal samples. The appropriate soft thresholding power was selected using the “pickSoft Threshold” function according to the standard of a scale-free network. Second, the Pearson correlation coefficient and corresponding *p* value were calculated for each gene pair (including lncRNAs and mRNAs) to construct the adjacency matrix by calculating the correlation matrix pairs. Finally, the topological overlap matrix and corresponding dissimilarity were transformed from the adjacency matrix. The hierarchical clustering was further built, and similar gene expressions were divided into different modules. Each module, which was assigned a unique color, contained a unique set of genes.

### LncRNA target gene and functional enrichment

The target genes of DELs were divided into the cis- and trans-target genes. To predict the *cis*-target genes, the genes located in the 100 kb upstream and downstream of DELs were screened using the LncTar software (Li et al., 2015). In contrast, the gene with a correlation coefficient >0.95 or < −0.95 with a DEL was considered to be the *trans*-target gene using the RNAplex software (Fatica and Bozzoni, 2014). Subsequently, Gene Ontology (GO) and Kyoto Encyclopedia of Genes and Genomes (KEGG) pathway enrichment were performed to determine the potential functions of the target genes of DELs and the DEDs and identify their participated pathways by the DAVID 6.8. A corrected *p*-value < 0.05 was used as the threshold to define the significant enriched GO terms or KEGG pathways.

### LncRNA-mRNA network construction

To further explore the interactions between the DELs and DEGs during rumen development, LncRNA-mRNA networks were constructed and visualized by Cytoscape v3.1.1 (Saito et al., 2012). The Pearson correlation coefficient (PCC) was calculated between DELs and DEGs. Only the DEL*-*DEG pair with an absolute value of PCC  >  0.95 and *p* < 0.01 were defined as co-expressed pairs and used to draw the networks of lncRNA-mRNA.

### Validation of RNA-sequencing by qPCR

To assess the reliability of the RNA Sequncing data, the quantitative PCR (qPCR) was used to determine the expressions of three randomly selected lncRNAs. cDNA was synthesized by the NovoScriptR Plus All-in-one 1st Strand cDNA Synthesis SuperMix (Novoprotein, Shanghai, China). Subsequently, qPCR was performed on a Bio-Rad cfx96 Touch Real-time PCR System (Bio-Rad, United States). The qPCR was performed in a 10 µl system, including 5 µl NovoStartR SYBR qPCR SuperMix Plus (Novoprotein), 0.4 µl each of forward and reverse primer (10 µM), 3.4 µl RNase-free ddH_2_O, and 0.8 µl cDNA. The qPCR reaction conditions are as follows: denaturation for 30 s at 95°C, followed by 40 cycles of 10 s at 95°C, 30 s at 56.9°C or 59.4°C for annealing, and 10 s at 95°C. A final melting program ranging from 65–95°C with an increment of 0.5°C and acquiring fluorescence after each step. All the primers were designed using Primer Premier 5.0 software (PREMIER Biosoft, Palo Alto, California, United States) and synthesized by Sangon Biotech (Shanghai) Co., Ltd. (Supplementary Table S1). The relative expression was estimated using the 2^−ΔΔCt^ method with the internal reference gene β-actin.

## Results

### Histomorphometric characteristics of Goat stomach

**FIGURE 1:**
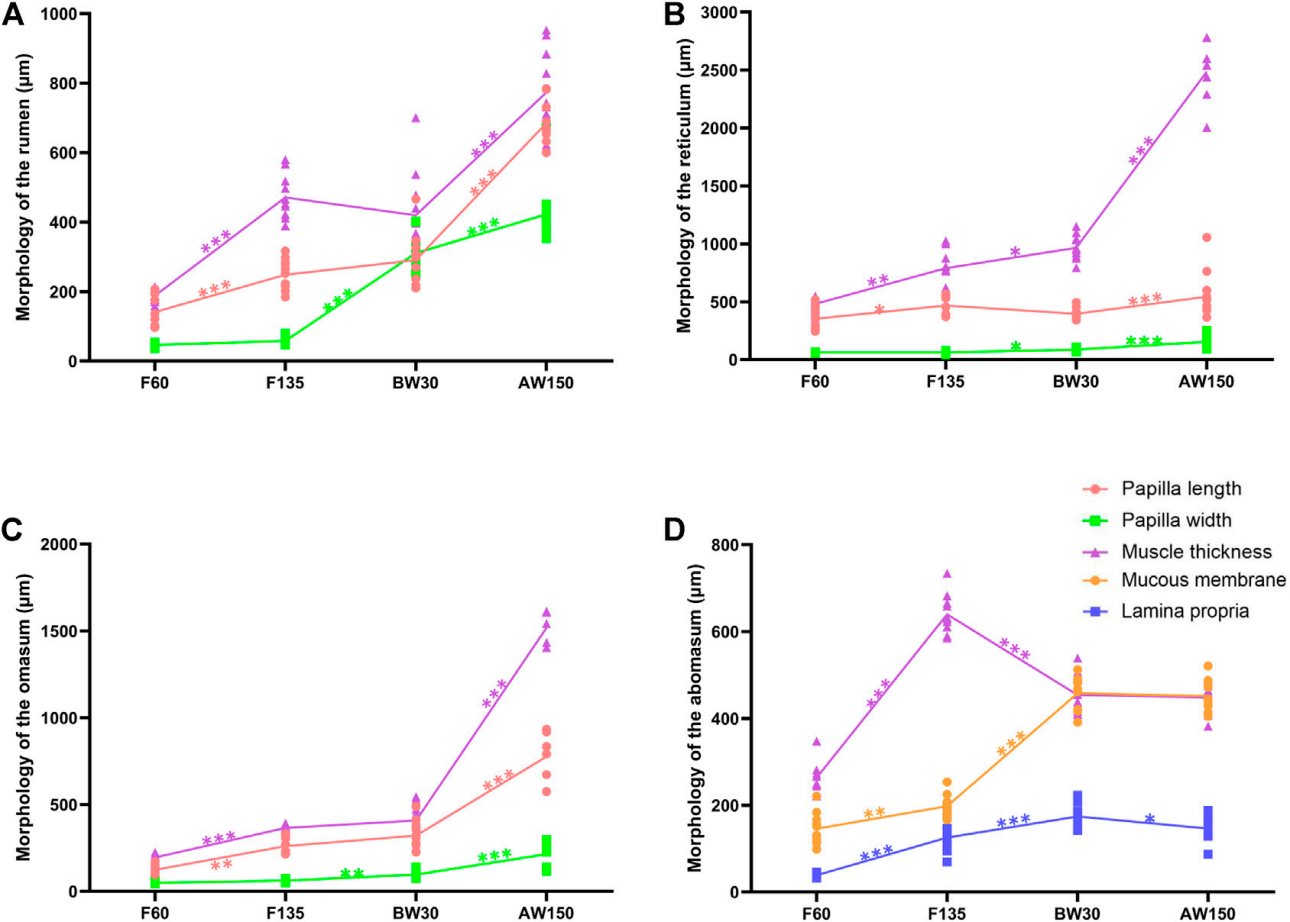
Morphometric analysis of goat stomach. Morphological determination of rumen **(A)**, reticulum **(B)**, omasum **(C)**, and abomasum **(D)** in goats.

At 60 days of gestation, the stratified epithelium was constituted by the stratum basale and stratum granulosum. Small ruminal papillae were observed protruding from the surface. The outline of the ruminal papillae (251.92 ± 44.08 μm) was evident at the embryonic 135 days (Figure 1A and Supplementary Figure S1). Compared to F60, the thickness of tunica muscularis (474.72 ± 64.55 μm) was significantly increased. In contrast, no significant difference was represented in the papillae width. At 30 days after birth, the width of the rumen papilla reached 298.69 ± 74.47 μm. At 150 days after birth, the growth of ruminal papillae was accelerated, and the length and width of the papillae were about five times and ten times that of F60. Morphological measurements showed that the papilla and muscular layer of the reticulum and omasum maintained a steady growth rate until birth and accelerated growth after weaning (Figures 1B,C). The thickness of the abomasum muscle at F60 was 149.70 ± 35.97 μm, and up to the maximum at F135, about three times that of F60 (Figure 1D). The thickness of the proprio layer and mucosa layer of the abomasum increased significantly from the fetal period to before weaning. However, the difference was not significant between before and after weaning periods, and it even decreased.

### Transcriptome profiles of lncRNAs and mRNAs in Goat rumens

**FIGURE 2:**
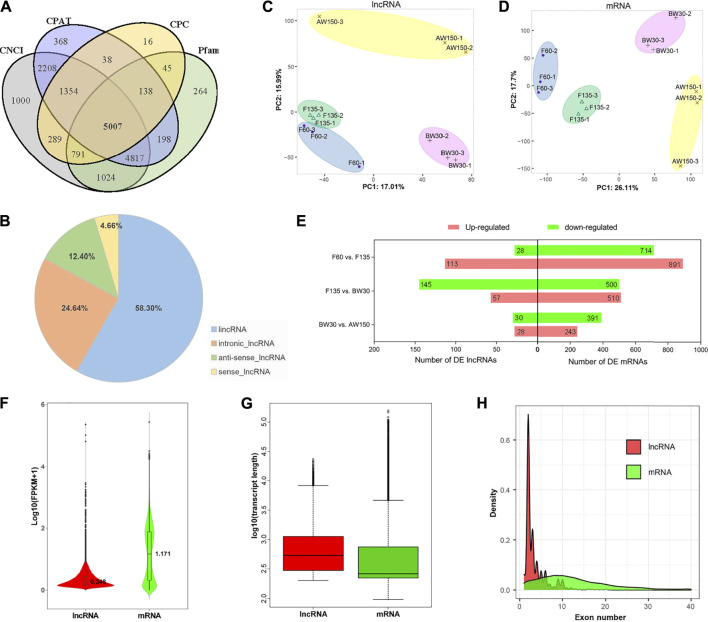
Properties of lncRNAs and mRNAs in goat rumen tissues. **(A)** Venn diagram of the lncRNAs either shared between or uniquely expressed in rumen tissues from different stages. **(B)** Prevalence of the different lncRNA types. **(C)** PCA of the lncRNAs. **(D)** PCA plot of the mRNAs. **(E)** Differentially expressed lncRNAs and mRNAs during goat rumen development. Red and green represent up-regulated and downregulated molecules, respectively. The expression level **(F)**, length distribution **(G)**, and exon number distribution **(H)** of lncRNAs and mRNAs. Red and green represent lncRNAs and mRNAs, respectively.

A total of 208.13 Gb clean data were generated in the twelve rumen tissues, representing the fetal stages (F60 and F135) and postnatal periods (BW30 and AW150, Supplementary Table S2). The comparison efficiency of reads from each sample with the goat reference genome ranged from 62.91% to 92.42% (Supplementary Table S2). After quality control, 5,007 lncRNAs were retained (Figure 2A, Supplementary Table S3), which classified into 2,919 intergenic lncRNAs (58.3%), 1,237 intronic lncRNAs (24.7%), 621 anti-sense lncRNAs (12.4%), and 230 sense lncRNAs (4.6%), respectively (Figure 2B). There were 19,738 mRNAs identified in the four developmental periods (Supplementary Table S4). The PCA plots revealed that both the lncRNAs and mRNAs clustered into four time-specific groups (Figures 2C,D). Furthermore, a total of 365 DELs and 2,877 DEGs were identified in the rumen tissues (Supplementary Tables S3,4), generating 141 DELs (113 up- and 28 downregulated) and 1,605 DEGs (891 up- and 714 downregulated) in F60 vs. F135, 202 DELs (57 up- and 145 downregulated) and 1,110 DEGs (510 up- and 500 downregulated) in F135 vs. BW30, 58 DELs (28 up- and 30 downregulated) and 634 DEGs (243 up- and 391 downregulated) in BW30 vs. AW150 groups (Figure 2E). In goat rumens, lncRNAs exhibited longer transcript lengths than mRNAs, while their expressions and exon number were lower than those of the identified mRNAs (Figures 2F–H).

### Temporal expression patterns of DELs and DEGs in Goat rumens

**FIGURE 3:**
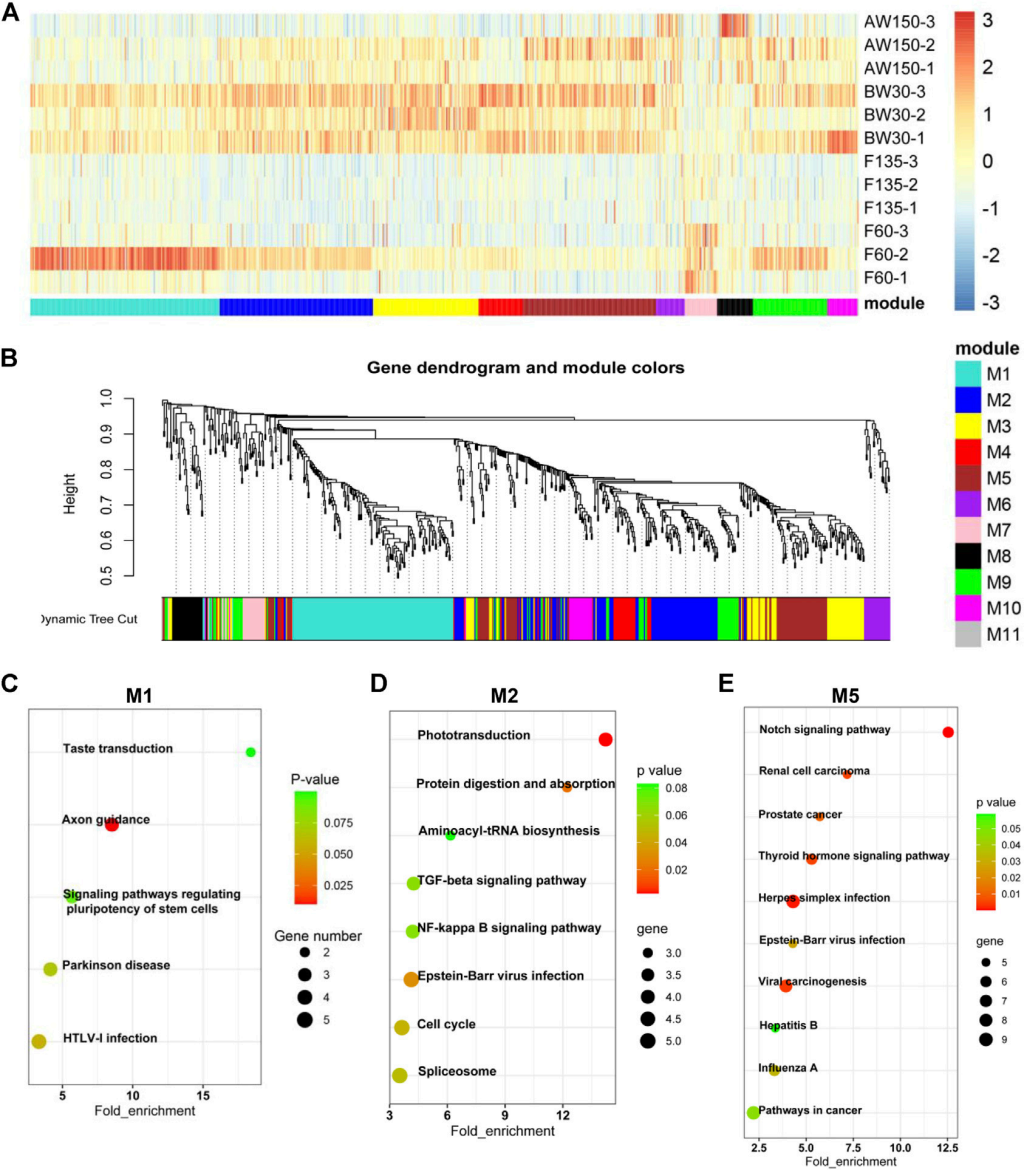
Dynamic expression of lncRNAs during goat rumen development. **(A)** Heatmap of the DELs during rumen development. **(B)** WGCNA co-expression network of the rumens. Modules of co-expressed genes were assigned a unique colour and number (M1-M11). **(C–E)** KEGG analysis of the DEGs in the M1 **(C)**, M2 **(D)**, and M5 **(E)** modules. Top 10 KEGG pathway enrichment terms. The horizontal axis represents a rich factor and the vertical axis represents the pathway. The size of the bubble indicates the number of target genes enriched in the pathway, and the color of the bubble represents a different *p*-value range. Fold enrichment is the ratio of DEGs numbers annotated in this pathway term to all gene numbers annotated in this pathway term.

**FIGURE 4:**
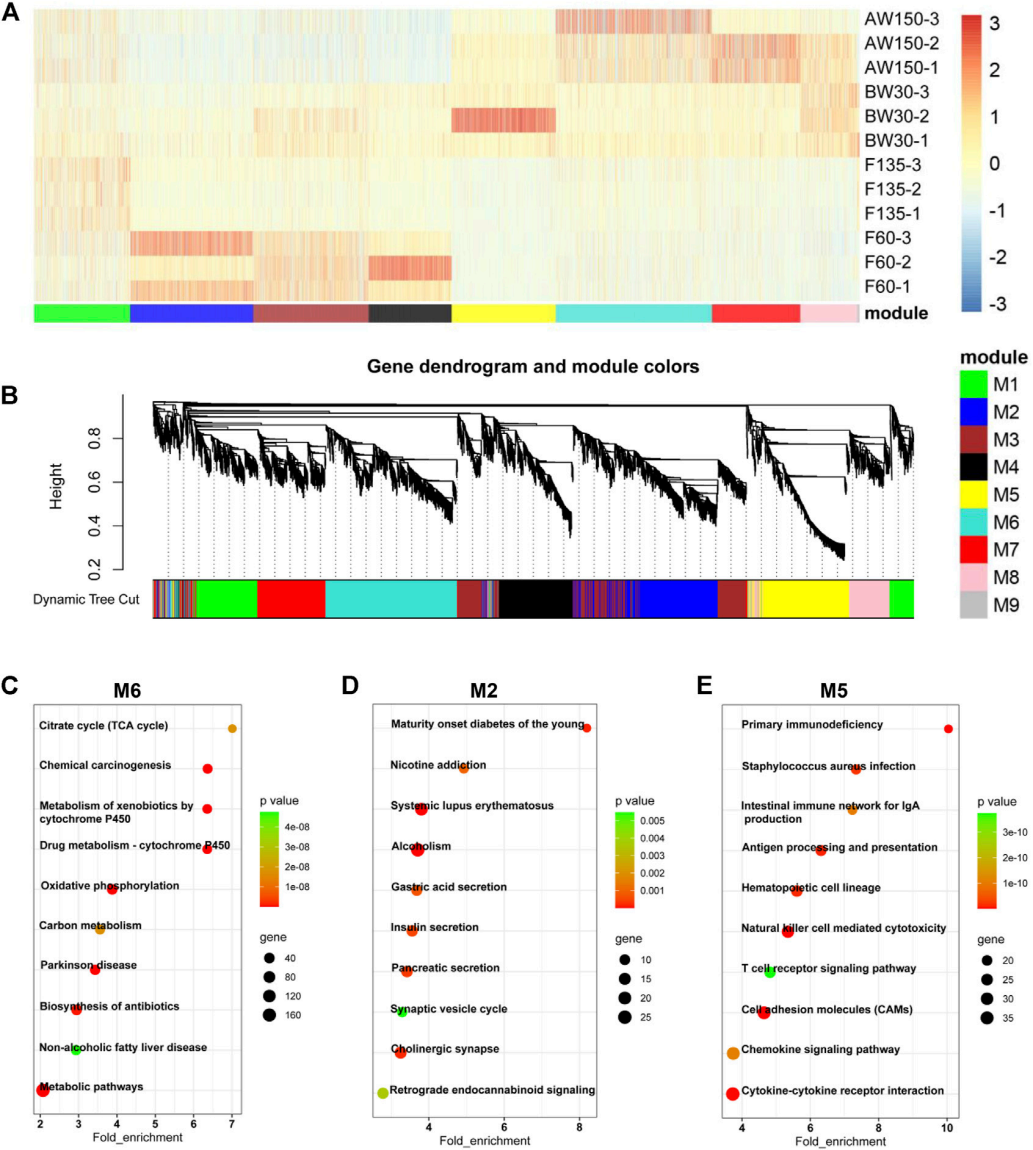
Dynamic expression of mRNAs during goat rumen development. **(A)** Heatmap of DEGs during rumen development. **(B)** WGCNA co-expression network analysis of the rumens. Modules of co-expressed genes were assigned a unique colour and number (M1-M9). **(C–E)** KEGG analysis of the DEGs in the M6 **(C)**, M2 **(D)**, and M5 **(E)** modules. Top 10 KEGG pathway enrichment terms. The horizontal axis represents a rich factor and the vertical axis represents the pathway. The size of the bubble indicates the number of target genes enriched in the pathway, and the color of the bubble represents a different *p*-value range. Fold enrichment is the ratio of DEGs numbers annotated in this pathway term to all gene numbers annotated in this pathway term.

To evaluate the prevalence and temporal profiles of DELs and DEGs in goat rumens, we performed the clustering and WGCNA analyses (Figures 3, 4). Eleven modules of DELs (M1–M11) were strongly correlated with the four developmental stages, and the modules (M1, M2, and M5) were abundantly expressed during the postnatal stages and up-regulated during rumen development (Figures 3A,B). KEGG analysis showed that the target genes of DELs in M1 were enriched for pathways related to disease and stem cell signaling (Figure 3C). The target genes in M2 were functionally involved in the cell cycle and immune response (Figure 3D). These target genes in M5 were significantly associated with cancer and cell proliferation (Figure 3E).

Accordingly, the expressions of DEGs showed a temporally dependent manner during the rumen development (Figure 4A) and harbored nine distinct co-expression modules (Figure 4B). The DEGs in M2 were mainly expressed during prenatal stages and gradually increased with rumen development. The DEGs in modules M5 and M6 were mainly expressed during the postnatal stages. The expressions of DEGs in M5 gradually decreased with rumen development, while they increased in M6. KEGG analysis showed that the DEGs in M6 were enriched for the pathways related to disease and cell metabolism (Figure 4C). The DEGs in M2 were functionally involved in the secretion of internal secretions and signal transduction (Figure 4D). These DEGs in M5 were significantly associated with cellular immunity (Figure 4E). In addition, the expressions of the three lncRNAs (MSTRG.78712.1, MSTRG.344984.4, MSTRG.27517.1) in M1, M2, and M4 were consistent with the results of RNA-sequencing (Supplementary Figure S2).

### Functional analyses of DELs involved in the rumen ontogenesis

**FIGURE 5:**
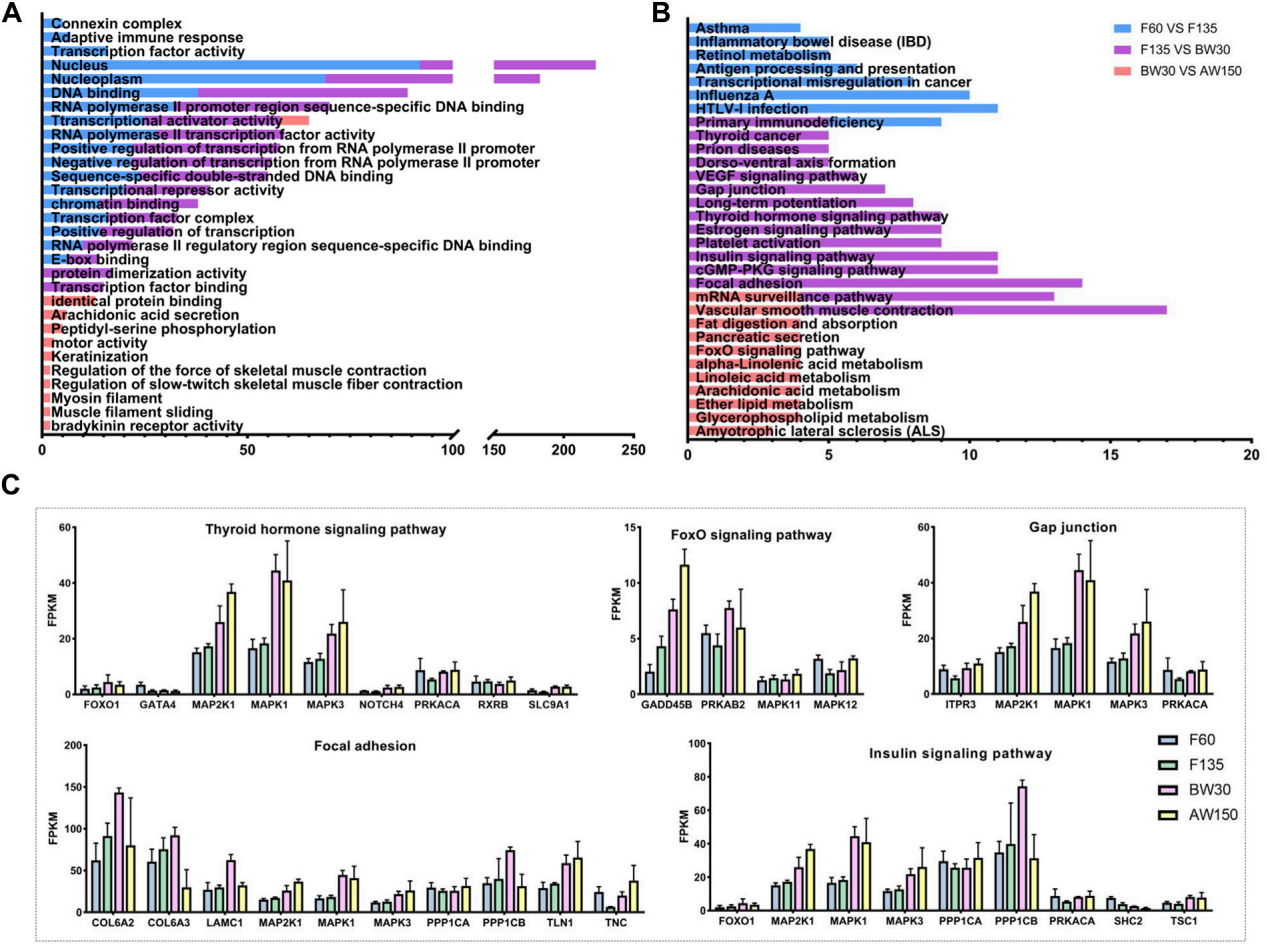
The GO and KEGG analysis of the target genes of DELs. **(A)** Top30 GO enrichment terms of the target genes of DELs. **(B)** Top 30 KEGG pathway enrichment terms of the target genes of DELs. Enriched GO terms and KEGG pathways associated with F60 vs. F135 are indicated in blue, F135 vs. BW30 are indicated in purple, BW30 vs. AW150 are indicated in pink. **(C)** The expression level of DELs in the key pathway.

Here, we identified the potential *cis*- and *trans*-target genes of the DELs and predicted their functions by GO and KEGG analyses. There were 236 *cis*-target genes predicted from the 141 DELs in F60 vs. F135, 382 *cis*-target genes identified in the 202 DELs in F135 vs. BW30, and 283 *cis*-target genes of the 58 DELs in the comparison of BW30 and AW150 (Supplementary Table S5). In addition, 468, 1,291, and 138 genes were identified in the DELs of the above comparisons in a *trans*-acting manner (Supplementary Table S5). GO showed that these target genes of DELs were significantly enriched in 117 terms (68 biological processes, 17 cellular components, and 32 molecular functions). The top enriched terms were DNA binding, nucleus, transcription factor complex, negative regulation of transcription, and positive regulation of transcription (Figure 5A and Supplementary Table S6). Importantly, some terms were related to rumen development and metabolism, such as adaptive immune response, keratinization, arachidonic acid secretion, and myosin filament. Subsequently, we detected the most significant enrichment of the 30 genes involved in the regulatory pathway (Figure 5B and Supplementary Table S7). Significantly, the majority of pathways are involved in cell development and biosynthesis. Among them, the target genes of DELs in the fetal period were mainly enriched in viral or disease-related signaling pathways. Primary immunodeficiency was enriched in F60 vs. F135 and F135 vs. BW30 groups. The target genes of DELs in the prenatal and postnatal stages were mainly enriched in focal adhesion, vascular smooth muscle contraction, cGMP-PKG signaling pathway, and insulin signaling pathway. Vascular smooth muscle contraction was also observed in BW30 vs. AW150. The target genes of DELs in the per- and post-weaning groups were mainly enriched in metabolism-related signaling pathways, such as alpha-linolenic acid metabolism, arachidonic acid metabolism, fat digestion and absorption, suggesting that lncRNAs play a role in gene regulation related to rumen development. The expressions of genes in the crucial pathways affecting rumen development are shown in Figure 5C. Notably, AMP-activated protein kinase (*AMPK*), Mitogen-Activated Protein Kinase (*MAPK1*), and Collagen Type VI (*COL6*) family genes were highly expressed in the key pathways, suggesting that these genes could be denoted as the candidate genes and act in essential roles during rumen development.

### Functional characteristics of DEGs related to Goat rumen ontogenesis

**FIGURE 6:**
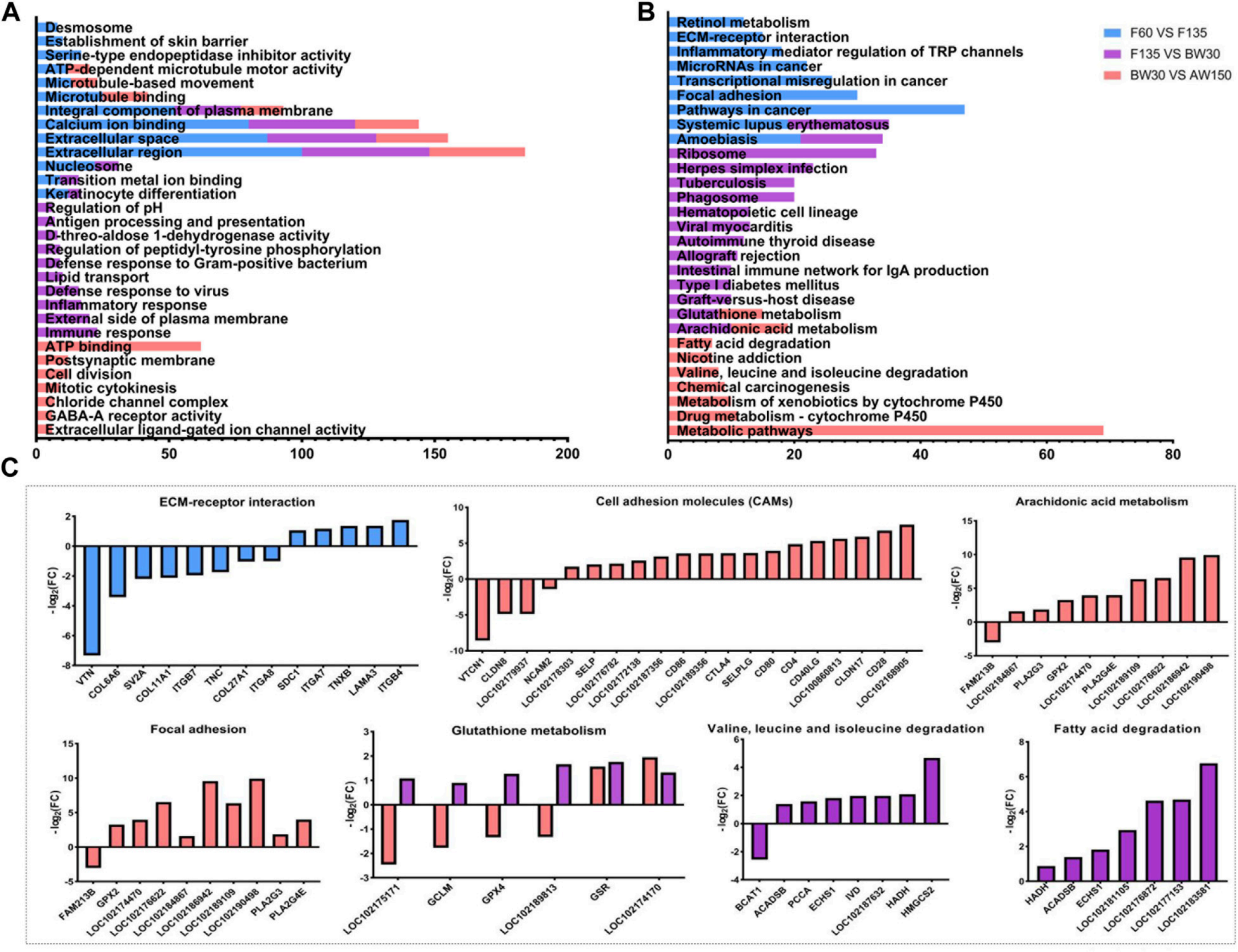
The GO and KEGG analysis of the DEGs. **(A)** Top 30 GO enrichment terms of the DEGs. **(B)** Top 30 KEGG pathway enrichment terms of the DEGs. Enriched GO terms and KEGG pathways associated with F60 vs. F135 are indicated in blue, F135 vs. BW30 are indicated in purple, BW30 vs. AW150 are indicated in pink. **(C)** The expression fold change of DEGs in the key pathway.

We found that the DEGs identified in the rumen tissues were significantly enriched in 290 stages (168 biological processes, 54 cellular components, and 68 molecular functions, Supplementary Table S8). In the molecular function category, DEGs were enriched for the terms with the establishment of the skin barrier, keratinocyte differentiation, regulation of pH, lipid transport, and microtubule-based movement in the rumens (Figure 6A and Supplementary Table S8). In the cellular component category, DEGs related to the extracellular region, extracellular space, and integral component of plasma membrane were enriched in the three comparisons (F60 vs. F135, F135 vs. BW30, and BW30 vs. AW150). In this way, DEGs were involved in the Transition metal ion binding, serine-type endopeptidase inhibitor activity, ATP-dependent microtubule motor activity, and extracellular ligand-gated ion channel activity in the biological process category. Significantly, DEGs were associated with Calcium ion binding were significantly enriched in the three groups, which may be important for rumen epithelial formation.

The top 30 KEGG pathways are shown in Figure 6B; Supplementary Table S9. Among them, there were 69 DEGs enriched in the metabolic pathways, followed by 47 DEGs in cancer pathways and 33 DEGs in the ribosome. Interestingly, unlike DELs, DEGs were mainly enriched in pathways related to human disease and immune response. Especially in F135 vs. BW30, DELs were involved in the ribosome, cell adhesion molecules, and arachidonic acid metabolism. Furthermore, the expressions of the DEGs in several key signaling pathways are shown in Figure 6B. In ECM-receptor interaction, sixty-two percent of DEGs were down-regulated. Most of these DEGs related to cell adhesion and arachidonic acid metabolism were significantly upregulated, while those involved in glutathione metabolism were down-regulated in F135 vs. BW30 group (Figure 6C). The DEGs were significantly enriched in metabolic pathways, valine, leucine, and isoleucine degradation, fatty acid degradation, and arachidonic acid metabolism during the postnatal stage. Most of these DEGs are significantly up-regulated (Figures 6B,C).

### The lncRNA-miRNA interaction network in Goat rumens

**FIGURE 7:**
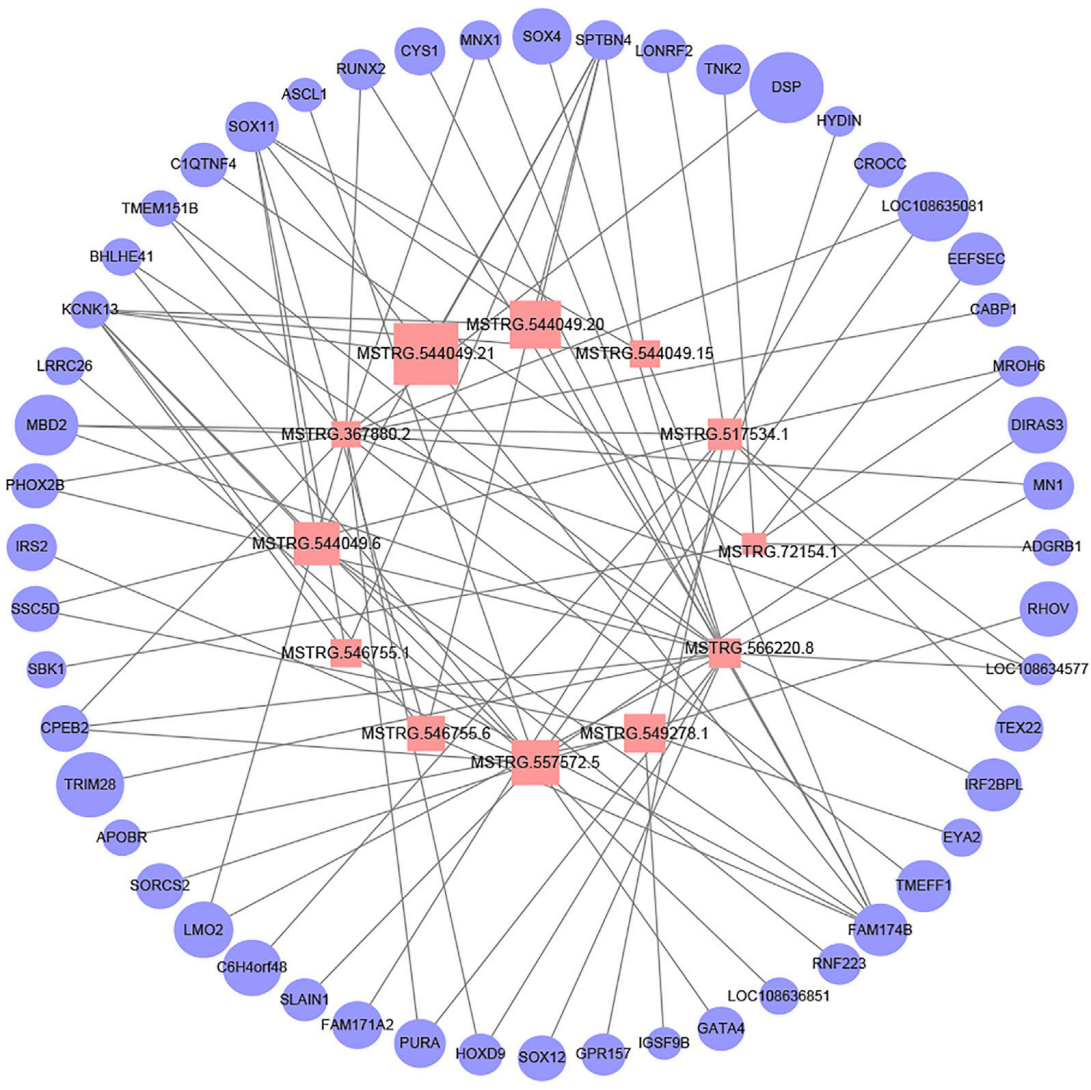
Network plot of candidate lncRNAs and mRNAs. Pink squares indicate lncRNAs and light blue circles indicate mRNAs. The graph size represents the FPKM of lncRNAs and mRNAs.

We constructed the lncRNA-mRNA regulatory network to further explore the possible interaction between lncRNA and mRNA during rumen development. A total of 90 DEL-DEG pairs were found to play a role in rumen development. The figure shows the predicted results, indicating that lncRNA may bind to mRNA during rumen development and play the role of molecular sponge to regulate rumen development (Figure 7). Except MSTRG.367880.2, all the other lncRNAs were differentially expressed in F135 vs. BW30. Notably, the candidate lncRNAs presumably regulated rumen development through *SPTBN4*, *SOX11*, *KCNK13*, and *FAM174B*. Notably, the differentially expressed target genes of multiple lncRNAs (MSTRG.566220.8, MSTRG.354575.1, MSTRG.549278.1, MSTRG.155742.1, and MSTRG.396834.25) were functionally enriched in the PI3K-Akt signaling pathway. In addition, MSTRG.566220.8 and MSTRG.367880.2 appear to regulate FoxO1, suggesting regulating cell cycle and apoptosis through the FoxO signaling pathway.

## Discussion

The rumen is the largest stomach chamber in adult ruminants. Approximately 80% of short-chain fatty acids, and many nutrients, such as ammonia, amino acids, small peptides, glucose, and inorganic ions, are absorbed and transported by the rumen epithelial cells. The morphological and functional development of rumen will directly affect the feed utilization rate and performance of goats (Carballo et al., 2019; Kent-Dennis and Penner, 2021). Until 60 days of gestation, the differentiation of the rumen wall was completed, and the rumen epithelium was divided into the basal and granular layers, accompanied by a slight eversion from the basal layer to the granular layer, forming the rumen papilla (Zhong et al., 2022). Similar to the previous study (García et al., 2012), these fully developed papillae reached the epithelial surface at 150 days of gestation, and the length of the ruminal papilla increased significantly. However, there was no significant difference in papilla width. The rumen has no ruminal function during the early stage of lactation, and the milk directly enters the abomasum through the oral cavity and esophagus (Schäff et al., 2018). The rumen papillae and muscular thickness increased significantly through the physical stimulation of rumen tissue morphological development as lambs began to be fed (Lane et al., 2000). Furthermore, the composition and characteristics of solid diets also affected the development of ruminal epithelium, ruminal papilla, and musculature to varying degrees (Shen et al., 2017; Xie et al., 2020). In addition to the effects of feed composition and characteristics, the significant changes in rumen morphological, functional metabolism, and microbial colonization during rumen development should be co-regulated by multiple genes and regulatory factors (Zhong et al., 2020; Zhong et al., 2022). Nevertheless, the molecular regulatory mechanisms of rumen ontogenesis are still limited.

Recent studies have shown that lncRNAs play an essential role in many biological processes by controlling gene expression through cis or trans mechanisms (Bhat et al., 2010). Hence, the function of lncRNA can be inferred from understanding its target genes. The *cis*-regulatory lncRNA has enhancer-like activity and promotes the expression of adjacent genes. *Cis*-prediction results showed 110 DELs around 346 genes, with distances less than 100 Kb. It is noteworthy that some *cis*-target genes are involved in rumen development in goats. *Carbonic Anhydrase 9* (*CA9*) is a predicted *cis*-target of lncRNA MSTRG.190830.1, which catalyzes the hydration of carbon dioxide and dehydration of bicarbonate and participates in pH regulation (Bondzio et al., 2011; Becker, 2020). More interesting, *CA9* was also involved in the regulation of cell proliferation and transformation and its precise functions in pH regulation and ion transport (Bondzio et al., 2011). *CA9* expression increased significantly with the increase of acid secretion capacity. In mouse gastric surface epithelial cells, CAIX ablation down-regulates Claudin-18, leading to sustained acid reflux down-regulation and disruption of gastric wall barrier function (Li et al., 2018). From the short review above, key findings emerge: *CA9* may be involved in regulating rumen epithelial cell proliferation and differentiation. A target of MSTRG.324173.1, Ets homologous factor (EHF) is a member of the epithelial-specific Ets (ESE) transcription factor family (Reehorst et al., 2021). EHF is highly compatible with the C-MET gene of the receptor encoding dispersion factor or hepatocyte growth factor, which is involved in epithelial differentiation (Kas et al., 2000). Importantly, it acts as an inhibitor of specific subsets of ETS/AP-1 responsive genes and as a regulator of nuclear responses to mitogen-activated protein kinase signaling cascades, regulating epithelial differentiation and proliferation (Tugores et al., 2001). In brief, it is inferred that may have roles in rumen development.

In this way, many lncRNAs still regulate target genes in a trans manner, and these target genes are far from the transcription site of lncRNA. Co-expression analysis revealed that 104 DELs interacted with 1,003 genes (r-value > 0.95 or < −0.95 and *p*-value < 0.05). KEGG analysis of target genes involved several key signaling pathways, including protein digestion and absorption, focal adhesion, Insulin signaling pathway, gap junction, FoxO signaling pathway, and Unsaturated fatty acid metabolism. Among them, MSTRG.566220.8 and MSTRG.367880.2 appear to regulate *FoxO1*, suggesting that they may regulate cell cycle and apoptosis through the FoxO signaling pathway. Moreover, multiple lncRNAs may regulate rumen development by targeting mRNAs (*COL4A4*, *COL6A2*, *TNC*, *COL6A3*, *MAPK1*, *TSC1*, *LAMC1*, *MAPK3*) on the PI3K-Akt signaling pathway. Mitogen-activated Protein Kinase 1 (*MAPK1*) is highly compatible with MSTRG.566220.8, which can be used as an integration point for multiple biochemical signals and participate in various cellular processes such as proliferation, differentiation, transcriptional regulation and development (Yoon and Seger, 2006). These aforementioned KEGG pathways have been suggested to be involved in rumen development. The urea transporter *SLC14A2* is predicted to be the target gene of lncRNA MSTRG.549278.1, which is responsible for transporting endogenous urea from the blood to the rumen lumen during the urea cycle, providing an additional nitrogen source for bacterial protein synthesis in the rumen (Stewart et al., 2005; Pan et al., 2021). Germline mutations of the breast cancer-associated gene 1 (*BRCA1*) are a predictive target of lncRNA MSTRG.396735.21. BRCA1 induces replication arrest to repair DNA damage by mediating *P21* and *GADD45* to activate cell cycle checkpoints (Yoshida and Miki, 2004). Loss of *BRCA1* leads to abnormal cell cycle checkpoints and triggers cell responses to DNA damage, preventing cell proliferation and inducing apoptosis (Deng, 2006; Takaoka and Miki, 2018). MSTRG.396735.21-*Notch 4* gene may be involved in the regulation of rumen development. The Notch 4 receptor is a single transmembrane protein encoded by the Notch gene. Notch receptor signaling between adjacent cells regulates cell differentiation, proliferation, and apoptosis (Kopan and Ilagan, 2009). These results suggest that these lncRNAs may be closely related to rumen development. Although these lncRNAs require further experimental studies, this information may help us to explore the potential regulatory mechanisms of lncRNAs involved in rumen development in goats.

## Conclusion

In the current study, we characterized the transcriptome of goat rumens and profiled the expressions of lncRNAs and mRNAs at four different developmental stages (F60, F135, BW30, and AW150). A total of 365 DELs and 2,877 DEGs were identified during rumen development. The cis- and trans-target genes of DELs were mainly enriched in pathways related to rumen cell growth and metabolism, representing a different manner of DEGs. In addition, we explored 87 lncRNA-mRNA molecules closely associated with goat rumen development and proposed the functional noded genes responsible for the regulation of rumen ontogenesis. Furthermore, the functions of these candidate genes need to be verified and explored in the rumen epithelium cells. Our study provides a valuable resource for yielding the lncRNA database of goats and is helpful for understanding the regulatory mechanism of rumen development in ruminants.

## Data Availability

The datasets presented in this study can be found in online repositories. The names of the repository/repositories and accession number(s) can be found below: Goat rumen RNA-Seq data are deposited in the SRA database (PRJNA720177).
