# Supplementary figures and images for: LncRNA-mRNA modules involved in goat rumen development: Insights from genome-wide transcriptome profiling
Source: Front Physiol. 2022 Aug 24;13:979121. doi: 10.3389/fphys.2022.979121 (PMC9449361; doi:10.3389/fphys.2022.979121)

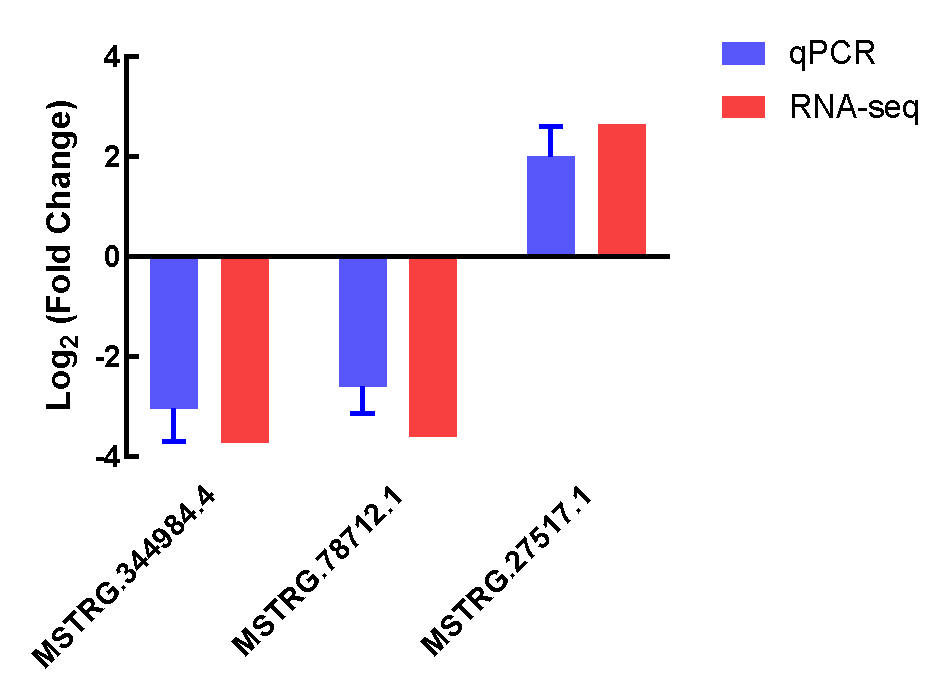

Supplement: Supplementary file 4 [file Image2.TIF]

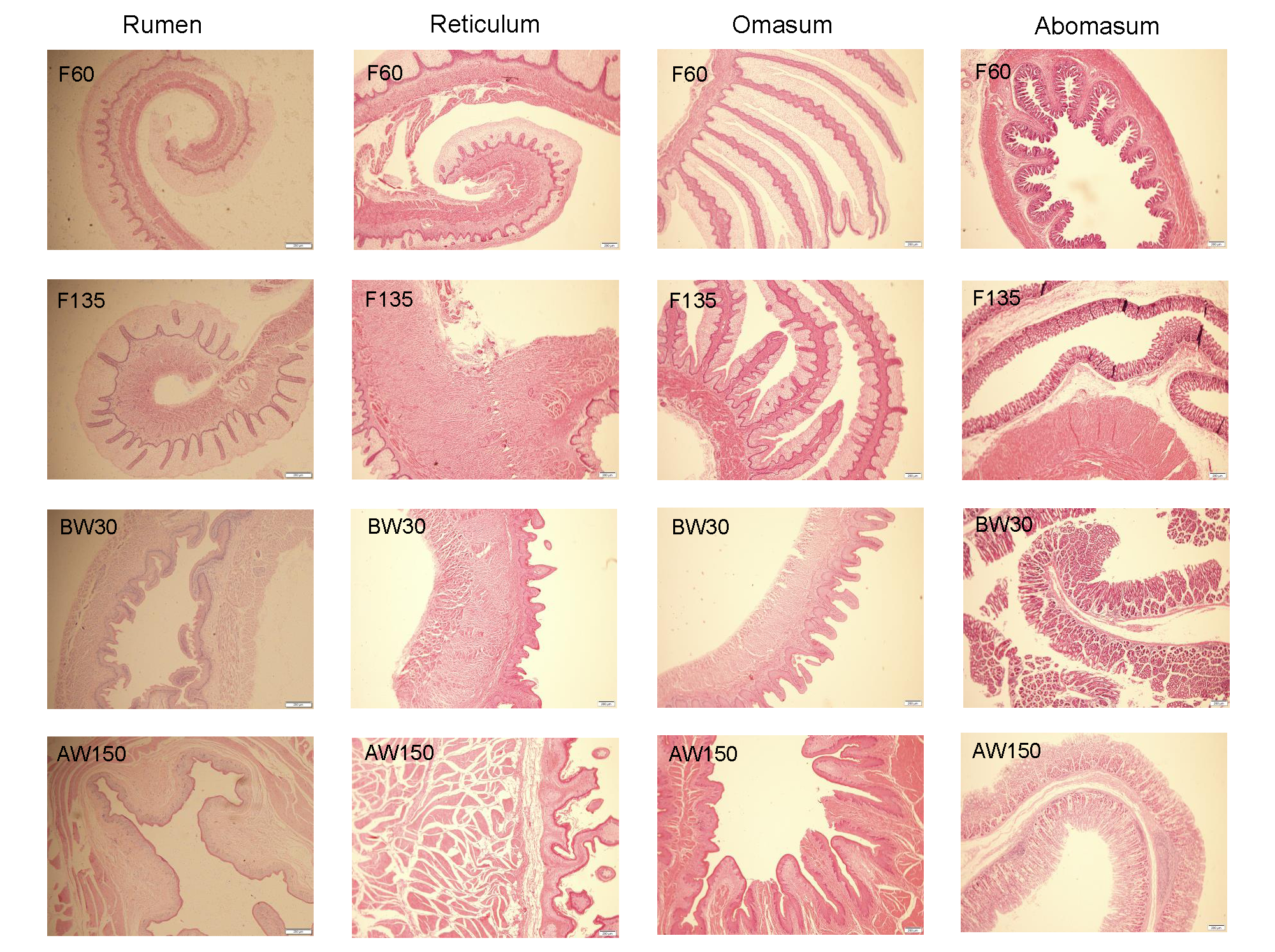

Supplement: Supplementary file 5 [file Image1.TIF]
